# Characterising mental health and organisational outcomes in Bristol’s night time economy workforce: a cross-sectional online survey

**DOI:** 10.1186/s12889-026-28676-4

**Published:** 2026-07-22

**Authors:** Yvanna Todorova, Aron Sherry, Denis M. Ngina, Denise Kendrick, Jo Leonardi-Bee, Lynn Stanley, Carly Heath, Lauren B. Sherar, Elizabeth Orton, Jane Bourke, Holly Blake

**Affiliations:** 1 School of Sport, Exercise and Health Sciences, Loughborough University, Loughborough, UK; 2 Centre for Academic Primary Care, School of Medicine, University of Nottingham, Nottingham, UK; 3 Centre for Evidence Based Healthcare, School of Medicine, University of Nottingham, Nottingham, UK; 4 Department of Economics, Bristol City Council, Bristol, UK; 5 NIHR Leicester Biomedical Research Centre, Leicester General Hospital, Gwendolen Road, Leicester, UK; 6 Cork University Business School, University College Cork, Cork, Ireland; 7 School of Health Sciences, University of Nottingham, Nottingham, NG7 2HA UK; 8 NIHR Nottingham Biomedical Research Centre, Nottingham University Hospitals NHS Trust, Nottingham, UK

**Keywords:** Workforce, Night Work, Mental Health, Hospitality, Organisations

## Abstract

**Background:**

Working at night poses several physiological and psychological barriers to mental health, which could influence organisational productivity through presenteeism (coming to work when ill) and turnover intention. Yet, the prevalence of poor mental health and its association with organisational outcomes is not clearly defined in the Night Time Economy (NTE).

**Methods:**

This cross-sectional online survey aimed to characterise and compare mental health (anxiety, depression, mental well-being, burnout) and organisational outcomes (presenteeism, work engagement and turnover intentions) in Bristol’s NTE hospitality workforce. Descriptive statistics and appropriate parametric and non-parametric tests were used to summarise and compare mental health and organisational outcomes. Multivariable linear and logistic regression explored association between wellbeing and organisational outcomes.

**Results:**

Over half of the 181 respondents (66% aged < 34 years, 54% female, from 44% small organisations) reported signs of depression and/or burnout, with a third of the respondents reporting moderate-to-severe anxiety. There were high levels of presenteeism (85%) and high turnover intentions (40%). Less than half of the respondents had sick pay and/or breaks during night work. Anxiety ([adjusted odds ratio] aOR = 4.00, 95% CI 1.59–10.65, *p* = 0.004), depression (aOR = 3.44, 95% CI 1.40–8.83, *p* = 0.008), and burnout (aOR = 2.85, 95% CI 1.17–7.36, *p* = 0.024) were associated with a higher likelihood of presenteeism. Anxiety ([adjusted mean difference] AMD = − 0.32, 95% CI − 0.64 to − 0.01, *p* = 0.043), depression (AMD = − 0.62, 95% CI − 0.91 to − 0.33, *p* < 0.001), poor wellbeing (AMD = − 0.79, 95% CI − 1.12 to − 0.52, *p* < 0.001), and burnout (AMD = − 0.60, 95% CI − 0.89 to − 0.32, *p* < 0.001) were associated with lower work engagement. Anxiety (aOR = 3.54, 95% CI 1.44–9.23,*p* = 0.007), depression (aOR = 2.70, 95% CI 1.14–6.67, *p* = 0.027), poor wellbeing (aOR = 4.84, 95% CI 2.12–11.74, *p* < 0.001), and burnout (aOR = 8.45, 95% CI 3.39–23.57,*p* < 0.001) were also associated with greater turnover intentions.

**Conclusions:**

Poor mental health is prevalent in the NTE workforce and is associated with presenteeism, work engagement, and turnover intentions. Given the high costs to organisations of presenteeism and turnover, a tailored mental health intervention is needed in the NTE.

**Supplementary Information:**

The online version contains supplementary material available at https://doi.org/10.1186/s12889-026-28676-4.

## Introduction

In 2023, the night-time economy (NTE), described as work centred around activities happening between 6 pm and 6 am [1, 2], accounted for 4.1% of the UK economy, contributing £93.7 billion [3, 4]. Night-time work differs from traditional shift work as the operation of business revolves around night-time activities. For example, bars and music venues mainly operate during night hours and are associated with a distinct focus on night time cultural and leisure activities (hereafter referred to as “hospitality”). With 23% of those NTE workers (452,000 jobs) working in restaurants, pubs, and entertainment venues [5], it is important to characterise and understand the unique mental health and organisational outcomes landscape of this distinct working population.

Little is known about the independent effects of NTE work on mental health and wellbeing [6]. The precarious nature of hospitality work is associated with presenteeism (working while ill), which is estimated to affect 38.3% of European hospitality workers [7]. Hospitality also records high turnover rates, mainly due to low pay, poor work-life balance and lack of job satisfaction [8]. However, evidence investigating the independent effects of NTE work on mental health and wellbeing is limited. Most existing research tends to focus on irregular or unpredictable shift patterns rather than examining night or evening work in isolation [1, 9–13]. In recent years, there have been some studies highlighting the connections between NTE work and mental health [14–16]; however, these focus on specific jobs such as hotel workers or chefs and fail to capture the impact of a broader ranges of NTE jobs, such as performing art work or events management, on mental health and how it interacts with organisational outcomes.

The aim of the study was to [1] characterise the prevalence of poor mental health (depression, anxiety, well-being and burnout) [2], characterise the prevalence of adverse organisational outcomes (presenteeism, poor work engagement, and turnover intention), and [3] describe any associations between demographic characteristics, poor mental health and organisational outcomes.

## Methods

### Study design and setting

The cross-sectional online survey was developed as part of a larger process evaluation [17] informed by the Reach, Effectiveness, Adoption, Implementation, and Maintenance (RE-AIM) [18] and Practical Implementation Sustainability Model (PRISM) evaluation frameworks [19]. Reporting was guided by the Strengthening the Reporting of Observational Studies in Epidemiology (STROBE) statement [20] (Supplemental File 1) and the Checklist for Reporting Results of Internet E-Surveys [21]. The setting was Bristol’s NTE hospitality sector (covering restaurants, music venues, pubs, hotels, festivals, event promotion, security, and freelance work). The study received approval from the University of Nottingham Faculty of Medicine and Health Sciences Research Ethics Committee (FMHS 192–0524, [July 2024]).

### Study population and sample size

Inclusion criteria were people aged 18 years and over, working in the NTE hospitality sector, and based in Bristol, UK. With an estimated population size of 116,000 nighttime workers in Bristol, a population proportion of 50%, a 5% margin of error and 95% confidence interval, the target sample size was 383.

### Study procedures

The survey was undertaken between August 2024 and January 2025. The participant information sheet outlining the study, informed consent form and survey were available via Qualtrics. All participants were required to provide informed consent before proceeding to the survey. The research team worked with the several distribution channels (e.g., social media (e.g., X [formerly Twitter], Facebook, LinkedIn), professional networks, emails, and distribution lists held by NTE leads at Bristol City Council and their communications partner organisation) to recruit Bristol-based NTE workers and team leaders via a media company to increase reach across occupational groups and settings and minimized sampling bias. There was an opt-in incentive of a £250 shopping voucher prize draw for those who fully completed the survey.

#### Public and patient (PPI) contribution

The Bristol Nights team, part of Bristol City Council, UK, conducted a sense check of the language used in the survey items to ensure it was appropriate for the NTE hospitality sector. A further six individuals working in the NTE (public contributors) reviewed the survey prior to administration to check appropriateness of content, usability, and technical functionality.

Terminology is important to this sector. Therefore, the term “worker” refers to staff, employees, zero-hour contract workers and freelancers within the NTE. The term “team leader” refers to workers within the NTE organisation that have any degree of management responsibilities.

### Data collection

Survey content was developed by the research team with occupational health expertise. The survey was used to collect quantitative data including responses on a Likert scale, sliding scale and categorical responses with non- response options (i.e., ‘not applicable’, ‘prefer not to say’), and qualitative data in free text responses. The selection of standardised measures was done in consultation with the PPI group. Due to concerns about the impact of the survey length on recruitment success, abbreviated versions of certain measures were used. There were a total 53 questions divided over four sections. As the wider evaluation outcomes will be reported elsewhere, the variables reported in the present paper are:

i. Sociodemographic (15 questions, all multiple choice).
  - Sociodemographic characteristics focused on age, gender, ethnicity, highest qualification, occupational group, length of service, employment setting and work pattern.
ii. Mental health and well-being (4 questions; all Likert scales).
  - The World Health Organisation – Five Well-being Index [WHO-5 [22] ],
  - Patient Health Questionnaire [PHQ-2 [23] ],
  - Generalized Anxiety Disorder Scale [GAD-7 [24] ].
  - Single item of Burnout from the Physician Work Life Scale [25].
iii. Organisational outcomes (4 questions: 2 multiple choice and 2 Likert scale.)
  - Copenhagen Psychosocial Questionnaire Version III [COPSOQ-III] short form [26].
  - Presenteeism (asked as “In your current role, do you ever go to work despite feeling unwell?”).
  - Dedication sub-scale of the 9-item Utrecht Work Engagement Scale [UWES-9, 3 items: DE2, DE3, DE4 [27] ].
  - Turnover intentions [as used in Blake et al., 2020 [28] ],

Scoring of each item is described in Supplementary File 2. Items were not randomised or alternated. Items were visible one section at a time and were conditionally displayed based on responses to other items, to reduce the number and complexity of the questions. Respondents were able to review and change their answers through a ‘Back’ button. Survey completion was anonymous, although respondents were given the option of providing an email address to be contacted for future research or to opt in to the prize draw.

### Data analysis

Data from the NTE-wide survey was analysed using R statistical software. The completion rate was defined as the ratio of the number of people who finished the survey divided by those who completed the first page. Only finished survey responses were included in the analysis. Descriptive statistics were reported as absolute values (counts) and relative frequencies (percentages) for (i) sociodemographic, (ii) mental health and well-being, and (iii) organisational outcomes. Sociodemographic and mental health and well-being descriptive statistics have been reported in a larger evaluation paper where these variables have been broken down by evaluation-specific groups: ‘engager’ and ‘non-engagers’ [17]. Histograms and the Shapiro-Wilk test were used to assess the normality of the scores and Levene’s test to assess the homogeneity of variance. Medians and interquartile ranges were reported for non-normally distributed data, while means and standard deviations were used for normally distributed data.

T-tests, ANOVA, and their non-parametric equivalents (Mann-Whitney U test and Kruskal-Wallis test) were used to compare well-being, depression, anxiety, burnout, psychosocial factors and arrangements. Chi-square (χ²) and Fisher’s Exact tests were used to explore associations between sociodemographic and work arrangement variables and organisational outcomes (presenteeism and turnover intentions).

A *post hoc* analysis was performed to assess the association between mental health measures and organisational outcomes: multivariable linear regression was used for continuous outcome (work engagement scores) and multivariable logistic regression was used for binary outcomes (turnover intentions and presenteeism). The models were only adjusted for gender, the highest level of education, age category and ethnicity as *a priori confounders*. The univariable models were built for each mental health measure, and the unadjusted measures of effect size were reported. The a priori confounders were then added in one step to each model, and the adjusted measures of effect size were reported. The models were checked for assumptions: normality of residuals, constant variance of the residuals and multicollinearity (using Generalised Variance Inflation Factor). Mean differences and odds ratios were reported together with 95% confidence intervals. Incomplete surveys were removed by listwise deletion. No completed surveys were excluded from analysis; n varied according to number of completers per item. Statistical significance was set at *p* < 0.05.

## Results

### Samples descriptives

A total of 181 respondents completed the NTE-wide survey (completion rate: 48% [181 completions out of 375 first-page completions]). Most respondents were between 25 and 34 years old (38%), White British ethnicity (83%) and female (54%; Supplemental File 3). The highest educational attainment ranged from GCSE-level (7%) to postgraduate degree (13%) with 41% having an undergraduate degree (Supplementary File 3).

88% of respondents worked in micro-to-medium organisations, most commonly bar or pub, music spaces, and restaurants (Table 1). 28% of respondents worked exclusively at night with the majority (65%) working a mix of day and night shifts. Only 37% of respondents worked in full-time employment. Nearly half (48%) of respondents were paid more than the UK living wage.

**Table 1:** Occupational Characteristics of the NTE-Wide Survey

Characteristic	Total *N* = 181	Percent (%)
Job Outside NTE Sector		
No	124	*68.5*
Yes	57	*31.5*
Employment Type		
Zero Hours	52	*28.7*
Part-time	32	*17.7*
Full time	68	*37.6*
Freelance/self-employed	29	*16.0*
Work shift Timing		
Night (between 6pm and 6am)	50	*27.6*
Day (between 6am and 6pm)	14	*7.7*
Both (An equal split of night and day)	52	*28.7*
Both: more night than day	36	*19.9*
Both: more day than night	29	*16.0*
Organisation or Venue Type (multiple options)		
Bar or pub	79	*43.7*
Event promoter	25	*13.8*
Music space	67	*37.0*
Hotel	1	*0.6*
Restaurant	40	*22.1*
Security Company	1	*0.6*
Festival	15	*8.3*
Other	22	*12.2*
Organisation Size		
Micro (less than 10 employees)	50	*27.6*
Small (10–50 employees)	79	*43.6*
Medium (50–249 employees)	31	*17.1*
Large (250 or more employees)	13	*7.2*
Don’t know	8	*4.4*
Hourly Pay		
Minimum wage	38	*21.0*
Living wage	35	*19.3*
More than living wage	87	*48.1*
Don’t know	6	*3.3*
Prefer not to say	15	*8.1*
Have a Team Leader		
Yes	137	*75.7*
No	44	*24.3*
Role (multiple options)		
Worker	81	*44.8*
Team leader/manager	100	*55.3*
Freelancer / contractor / self-employed	46	*25.4*
Specific role (multiple roles)		
Bartender	64	*35.4*
Bar manager	41	*22.7*
Waiting staff	30	*16.6*
Chef / kitchen staff	13	*7.2*
Restaurant manager	19	*10.5*
Security staff	4	*2.2*
Musician / artist / DJ	22	*12.2*
Hotel manager	0	*0.0*
Cleaning staff	6	*3.3*
Technician (e.g., sound/lighting)	10	*5.5*
Event team member	27	*14.9*
Event manager	39	*21.6*
Front of house manager	20	*11.1*
Promoter	25	*13.8*
Other	45	*24.9*

The mean well-being of the sample was a WHO-5 score of 45 (20.9) (Table 2), which is below the threshold for good mental health of 50 (Supplementary File 2). Nearly two thirds (64%) of the sample had signs of depression. Over a third (37%) of the sample reported moderate-to-severe anxiety (Table 2). Self-perceived burnout affected more than half (55%) of the overall sample (Table 2), with a third of respondents reporting “never” or “rarely” having a break during night work (Table 3). Nearly 76% of all respondents reported experiencing poor mental health in the workplace at some point, with only 34% of respondents having access to sick pay. Presenteeism was widespread with nearly 85% of respondents reporting they “sometimes” or “often” worked despite being unwell. Work engagement was moderate (Table 2). 40% reported that poor mental health had led them to consider leaving their job (turnover intention; Table 3).

**Table 2:** Standardised Mental Health and Well-being and Organisational Outcomes from the NTE-Wide Survey

Outcome	Total *N* = 181
GAD-7 score *(median*,* IQR)*	7.0 (8.0)
GAD-7 grouping *(N*,* %)*	
Minimal anxiety	64 (35.4)
Mild anxiety	50 (27.6)
Moderate anxiety	38 (21.0)
Severe anxiety	29 (16.0)
WHO-5 (%) Score *(mean*,* SD)*	45.6 (20.9)
WHO-5 grouping *(N*,* %)*	
Good well-being (score > 50)	74 (40.9)
Poor well-being (score < = 50)	107 (59.1)
PHQ-2 Score *(median*,* IQR)*	2.0 (3.0)
PHQ-2 grouping *(N*,* %)*	
Signs of depression (Score > = 2)	115 (63.5)
No signs of depression (Score < 2)	66 (36.5)
COPSOQ-III *(Mean*,* SD)*	
Quantitative Demands	47.6 (24.2)
Work Pace	69.1 (20.4)
Influence at Work	52.7 (26.6)
Recognition	61.4 (20.4)
Quality of Leadership	53.2 (24.5)
Mutual Trust Between Employees (Horizontal Trust)	74.3 (20.3)
Trust Regarding Management (Vertical Trust)	68.9 (20.2)
Justice at Work (Organisational Justice)	64.0 (22.7)
Social Support from Colleagues	58.2 (29.2)
Social Support from Supervisors	56.6 (31.0)
UWES-3 Score *(Mean*,* SD)*	
Work Engagement- Dedication	3.43 (1.0)
Physician Work Life Scale *(Median*,* IQR)*	
Burnout	3.0 (2.0)
Physician Work Life Scale *(N*,* %)*	
1 or more burnout signs	99 (54.7)
No burnout signs	82 (45.3)
Turnover intentions (*n* = 138) (n, %)	
No	65 (47.1)
Yes	73 (52.9)
Ever experienced mental health at work *(N*,* %)*	
No	43 (23.8)
Yes	138 (76.2)
Colleagues experienced mental-ill health at work *(N*,* %)*	
No	23 (12.7)
Yes	158 (87.3)
Presenteeism (work despite unwell) *(N*,* %)*	
Never	9 (5.0)
Rarely	19 (10.5)
Sometimes	74 (40.9)
Often	79 (43.7)

This table summarises well-being, mental health, work engagement, burnout, presenteeism, turnover intentions, and work psychosocial risk factors

The prevalence in the whole sample is also reported

*SD* standard deviation, *IQR* Interquartile Range, *COPSOQ-III* Copenhagen Psychosocial Questionnaire Version III. Used for work psychosocial risk assessment, *UWES-3* Utrecht Work Engagement Scale (UWES). Used to measures work dedication one of the core elements of work engagement in the tool

**Table 3:** Working Conditions and Turnover Intentions

Characteristic	Total *N* = 181
Break during nightwork (N, %)	
Never	19 (10.5)
Rarely	41 (22.7)
Sometimes	65 (35.9)
Often	32 (17.7)
Always	24 (13.3)
Typical Working hours (NTEHS role)	
Weekly Hours (mean, SD)	30.95 (14.9)
Length of Time in Current Role	
Years (SD)	5.24 (6.0)
Entitled to sick pay (N, %)	
No	92 (50.8)
Yes	61 (33.7)
Don’t know	28 (15.5)
Work despite feeling unwell *(N*,* %)*	
Never	9 (5.0)
Rarely	19 (10.5)
Sometimes	74 (40.9)
Often	79 (43.7)
Ever experienced poor mental health in current job role *(N*,* %)*	
No	43 (23.8)
Yes	138 (76.2)
Considering leaving or stopping job because of mental-ill health (*n* = 138)	
No	65 (35.9)
Yes	73 (40.3)
Colleagues experienced mental-ill health at work *(N*,* %)*	
No	23 (12.7)
Yes	158 (87.3)

*SD* standard deviation, *NTEHS* Nighttime economy hospitality sector, N for “Considering leaving or stopping job because of mental-ill health” was 138 as it was a filter question for those responding “Yes” to “ever experienced poor mental health in current job role”

### Associations between mental wellbeing and organisational outcomes

The survey data suggested that working conditions were associated with respondents’ mental health and well-being outcomes. Those who did not report entitlement to sick pay reported significantly higher levels of anxiety, but not depression, overall well-being, and work engagement (Table 4). Similarly, respondents who reported working while unwell (presenteeism) experienced significantly lower well-being and higher levels of both depression and anxiety (Table 4). Showing signs of burnout was also associated with significantly higher depression and anxiety levels and lower work engagement. Respondents who had ever experienced poor mental health at work reported poorer well-being, higher psychological distress, and lower work engagement. Turnover intentions due to mental health challenges displayed a clear pattern of poorer outcomes, with significantly higher anxiety and depression levels, lower well-being, and reduced work engagement (Table 4).

**Table 4:** Associations between Work Arrangements and Mental Health and Well-being

Characteristic	Well-being (WHO-5) Mean (SD)	*p*-value	Depression (PHQ-2) Median (IQR)	*p*-value	Anxiety (GAD-7) Median (IQR)		*p*-value	Work Engagement- Dedication (UWES-3) Mean (SD)	*p*-value	
Entitled to sick pay										
Yes	47.1 (21.6)	0.51^b^	2.0 (2.0)	0.47^d^		6.0 (7.0)	0.03^d^	3.6 (0.9)		0.12^b^
No	45.9 (21.0)		2.0 (3.0)			7.0 (10.0)		3.4 (1.1)		
Don’t know	41.6 (18.8)		2.0 (2.0)			10.5 (7.5)		3.1 (0.1)		
Work despite unwell										
Never/Rarely	56.3 (19.5)	0.01^a^	0.0 (2.0)	0.01^c^		3.5 (7.3)	0.01^c^	3.5 (0.9)		0.10^a^
Sometimes/Often	43.7 (20.6)		2.0 (2.0)			7.0 (8.0)		3.4 (1.0)		
Break during night work										
Never/Rarely	41.4 (20.8)	0.15^b^	2.0 (2.0)	0.12^d^		7.5 (7.5)	0.07^d^	3.2 (1.0)		0.19^b^
Sometimes	47.1 (20.6)		2.0 (3.0)			8.0 (11.0)		3.5 (1.0)		
Often/always	48.2 (20.8)		2.0 (3.0)			5.0 (8.0)		3.5 (1.0)		
Employment type										
Zero hours	40.54 (19.1)	0.04^b^	3.0 (2.0)	0.01^d^		8.5 (11.00)	0.02^d^	3.2 (1.1)		0.01^b^
Parttime	44.0 (18.9)		2.0 (2.0)			7.5 (9.25)		3.1 (0.9)		
Fulltime	46.5 (21.6)		2.0 (2.25)			5.0 (6.25)		3.5 (1.0)		
Self-employed	54.3 (22.1)		1.0 (3.0)			9.0 (11.00)		4.0 (0.8)		
Considering leaving job										
No	48.1 (19.9)	< 0.01^a^	2.0 (3.0)	0.01^c^		6.0 (6.0)	0.01^c^	3.6 (0.9)		< 0.01^a^
Yes	35.1 (17.5)		3.0 (2.0)			10.0 (9.0)		3.0 (0.9)		
Burnout										
Yes (1 or more signs)	35.1 (16.5)	< 0.01^a^	3.0 (2.0)	< 0.01^c^		10.0 (8.0)	< 0.01^c^	3.1 (1.0)		< 0.01^a^
No (No signs)	58.3 (18.4)		1.0 (2.0)			4.0 (6.0)		3.8 (0.8)		
Ever experience poor mental health										
No	59.7 (18.1)	< 0.01^a^	0.0 (1.5)	< 0.01^c^		3.0 (6.0)	< 0.01^c^	4.0 (0.9)		< 0.01^a^
Yes	41.2 (19.7)		2.0 (1.75)			8.0 (9.0)		3.3 (1.0)		
Colleague experience mental health health										
No	54.3 (21.2)	0.03^a^	1.0 (2.0)	0.04^c^		7.0 (12.0)	0.97^c^	4.1 (0.7)		< 0.01^a^
Yes	44.3 (20.6)		2.0 (2.0)			7.0 (8.0)		3.3 (1.0)		

The table summarises measures of well-being, depression, anxiety and work dedication for the full sample based on different work arrangements (*N* = 181)

Tests; a – t-test, b – Anova, c - Mann-Whitney U test, d - Kruskal- Wallis test, SD; standard deviation

Self-employed individuals tended to report better outcomes, with the highest levels of well-being and work engagement, and the lowest depression scores, whereas zero-hours and part-time workers showed lower well-being and work engagement (Table 4). Taking breaks from night work did not have a significant association with levels of anxiety, depression, or overall well-being. Age, contract type, and working times were associated with work engagement. Age and having breaks during work were also associated with turnover intentions (Table 5).

**Table 5:** Association of Demographics and Work Arrangements with Organisational Outcomes

Characteristic	Work Engagement- Dedication		Work despite unwell (presenteeism)			Turnover intentions		
	(uwes-3) Mean (SD)	*p*-value	Never/Rarely (*N*, %)	Sometimes/Often (*N*, %)	*p* - value	No (*N*, %)	Yes (*N*, %)	*p* - value
Age								
Under 25	3.0 (1.1)	< 0.01^b^	9 (18.0)	41 (82.0)	0.30^c^	17 (45.9)	20 (54.1)	0.04^c^
25–34	3.4 (1.0)		7 (10.1)	62 (89.9)		24 (38.7)	38 (61.3)	
35–44	3.8 (0.8)		5 (14.7)	29 (85.3)		18 (72.0)	7 (28.0)	
Over 45	3.8 (0.6)		7 (25.0)	21 (75.0)		6 (42.9)	8 (57.1)	
Gender								
Male	3.5 (1.1)	0.92^b^	13 (18.6)	57 (81.4)	0.16^c^	24 (49.0)	25 (51.0)	0.90^c^
Female	3.4 (0.9)		11 (11.3)	86 (88.7)		36 (46.8)	41 (53.2)	
Other	3.5 (0.9)		4 (28.6)	10 (71.4)		5 (41.7)	7 (58.3)	
Highest Education								
Low	3.5 (1.1)	0.89^b^	2 (15.4)	11 (84.6)	0.35^c^	7 (70.0)	3 (30.0)	0.27^c^
Medium	3.4 (1.0)		14 (20.3)	55 (79.7)		21 (42.0)	29 (58.0)	
High	3.4 (1.0)		12 (12.1)	87 (87.9)		37 (47.4)	41 (52.6)	
Student status								
No	3.5 (0.9)	0.45^a^	24 (15.6)	130 (84.4)	1.00^d^	56 (46.3)	65 (53.7)	0.61^c^
Yes	3.3 (1.1)		4 (14.8)	23 (85.2)		9 (52.9)	8 (47.1)	
Living arrangement								
Alone	3.6 (1.1)	0.32^b^	3 (12.5)	21 (87.5)	0.90^c^	10 (50.0)	10 (50.0)	0.64^c^
With partner/family	3.5 (0.9)		12 (15.6)	65 (84.4)		20 (41.7)	28 (58.3)	
With peers/Other	3.3 (1.0)		13 (16.2)	67 (83.8)		35 (50.0)	35 (50.0)	
Contract type								
Zero Hours	3.2 (1.1)	< 0.01^b^	7 (13.5)	45 (86.5)	0.47^d^	23 (54.8)	19 (45.2)	0.06^c^
Part-time	3.1 (0.9)		8 (25.0)	24 (75.0)		4 (20.0)	16 (80.0)	
Full time	3.5 (1.0)		9 (13.2)	59 (86.8)		27 (48.2)	29 (51.8)	
Freelance/self-employed	4.0 (0.8)		4 (13.8)	25 (86.2)		11 (55.0)	9 (45.0)	
Work timing								
Night (between 6pm and 6am)	3.1 (0.9)	0.04^b^	8 (16.0)	42 (84.0)	0.09^d^	18 (50.0)	18 (50.0)	0.88^d^
Day (between 6am and 6pm)	4.0 (0.6)		4 (28.6)	10 (71.4)		3 (60.0)	2 (40.0)	
Both (An equal split of night and day)	3.5 (1.0)		5 (9.6)	47 (90.4)		20 (45.5)	24 (54.5)	
Both: more night than day	3.2 (1.1)		3 (8.3)	33 (91.7)		14 (41.2)	20 (58.8)	
Both: more day than day	3.8 (0.9)		8 (27.6)	21 (72.4)		10 (52.6)	9 (47.4)	
Sick pay								
Yes	3.6 (0.9)	0.12^b^	10 (16.4)	51 (83.6)	0.87^c^	23 (46.9)	26 (53.1)	0.58^c^
No	3.4 (1.1)		13 (14.1)	79 (85.9)		30 (44.1)	38 (55.9)	
Don’t know	3.1 (0.9)		5 (17.9)	23 (82.1)		12 (57.1)	9 (42.9)	
Break during night work								
Never/Rarely	3.2 (1.0)	0.19^b^	6 (10.0)	54 (90.0)	0.20^c^	17 (33.3)	34 (66.7)	0.04^c^
Sometimes	3.5 (1.0)		14 (21.5)	51 (78.5)		27 (56.2)	21 (43.8)	
Often/Always	3.5 (1.0)		8 (14.3)	48 (85.7)		21 (53.8)	18 (46.2)	

*SD* standard deviation

For analysis, Gender was collapsed into 3 categories (male, female and other). Highest Education was collapsed into three categories, Low (“None of the above”, “GCSEs or equivalent”), Medium (“A/AS levels”, “NVQ or HND or HNC”, “Other professional qualification”) and High (“College or University degree”, “Postgraduate degree”). Living arrangement was collapsed into three categories, ‘Alone’ (living alone), ‘With partner/family’ (living with a partner or with family), and ‘With peers/other’ (living with friends, housemates, or other arrangements). Categories were collapsed to ensure sufficient sample size for statistical tests. Tests; a – t-test, b – Anova, c – Chi Square test, d – Fisher’s Exact test

In the *post hoc* analysis, poor mental well-being was consistently associated with negative organisational outcomes (Supplementary File 4). Individuals with signs of depression had, on average, work engagement scores that were 0.62 lower [ AMD − 0.62, 95% CI: -0.91, -0.33; *p* < 0.001] than those with no signs of depression. Some level of anxiety was associated with an average of 0.32 lower work engagement scores [AMD − 0.32, 95% CI: -0.64, -0.009; *p* = 0.043] compared to those with no or minimal anxiety. Poor well-being was also associated with lower work engagement, with scores averaging 0.79 lower [AMD − 0.79 95% CI: -1.06, -0.51; *p* < 0.001] than those of individuals reporting good well-being. Having one or more signs of burnout was associated with an average of 0.60 lower work engagement scores [AMD − 0.60, 95% CI: -0.88, -0.32; *p* < 0.001] compared to those with no signs of burnout.

Those with signs of depression were 3.4 times more likely to work sometimes or often despite being unwell [aOR 3.44, 95% CI: 1.40, 8.83; *p* = 0.008], compared to those with no signs of depression. Individuals with some level of anxiety were 4 times more likely to work while unwell, compared to those with no or minimal anxiety [aOR 4.00, 95% CI: 1.59, 10.65; *p* = 0.004]. Those with one or more signs of burnout were nearly 3 times more likely to work despite being unwell, compared to those with no signs of burnout [aOR 2.85, 95% CI: 1.17, 7.36; *p* = 0.024]. Overall well-being was not associated with presenteeism [aOR 1.68, 95% CI: 0.71, 4.07; *p* = 0.240].

Those with signs of depression were 2.7 times more likely to leave their roles compared to those with no signs of depression [aOR 2.70, 95% CI: 1.14, 6.67; *p* = 0.027]. Individuals with some level of anxiety were 3.5 times more likely to leave their role compared to those with no or minimal anxiety [aOR 3.53, 95% CI: 1.44, 9.23; *p* = 0.007]. Those reporting poor overall well-being were nearly 5 times more likely to leave their role compared to those with good well-being [aOR 4.84, 95% CI: 2.12, 11.74; *p* < 0.001]. Similarly, those with one or more signs of burnout were 8 times more likely to leave their roles compared to those with no signs of burnout [aOR 8.45, 95% CI: 3.39, 23.57; *p* < 0.001].

There was no evidence of problematic multicollinearity among predictors (all adjusted GVIF values < 1.1; highest = 1.07).

## Discussion

This cross-sectional study aimed to characterise and compare mental health and organisational factors within a UK Night Time Economy (NTE) hospitality workforce using validated assessment tools. To our knowledge, this is the first UK study to explore mental health and organisational outcomes within this sector. Analysis of survey data revealed that poor mental health outcomes (e.g., depression, anxiety, burnout) and poor organisational outcomes (e.g. presenteeism, turnover intentions) were highly prevalent. Furthermore, poor mental health indicators were consistently associated with adverse organisational outcomes, which has important implications for businesses within this industry.

Across all indicators, poor mental health outcomes were highly prevalent in this studied population. This included standardised assessments of depression, anxiety, and overall mental well-being, and well as poor mental health at work experienced personally and observed in others. While mental health is a concern across workplaces, several work characteristics within the NTE, such as irregular workload and fast-pace nature of organizations in the urban setting, were identified that may contribute to these findings [29]. This study identified additional factors that could potentially contribute to poor mental health; the sample consisted of workers from small-to-medium sized organisations as either part-time or zero-hour contract (precarious) workers. Small-to-medium organisations tend to have fewer mental health and well-being resources available for their workers, potentially increasing workers’ vulnerability to poor mental health [30]. Additionally, non-full-time work, characterised by uncertain time management and income, impacts economic, socio-relational, behavioural and physical factors that increase stress, anxiety and depression [31].

Poor occupational and organisational outcomes were also highly prevalent within this sample. High rates of presenteeism and turnover intention were accompanied by undesirable occupational factors including low rates of sick pay entitlement and high rates of limited or no worktime breaks. NTE workers perceived their workplace as fast paced with little to no breaks or sick pay. Fast paced work environments can contribute to a broad range of psychosocial work factors, such as higher perceived work demand, imbalance of effort and rewards, and less time for recovery, that can contribute to burnout, and poor overall wellbeing [32]. Minimal breaks during work may exacerbate burnout and poor mental health factors by reducing the individual’s ability to recover from stress and decrease work performance [33]. Lack of sick pay can also mean an increase in presenteeism, with prolonged sickness as well as decreased work performance [7, 34]. A systematic review suggested that improving mental health has three-times greater impact on absenteeism than physical health [35], highlighting that mental health is an essential component to addressing absenteeism and presenteeism. The results of this paper highlight that NTE work has a number of attributes (e.g. commonly small-to-medium sized organisations that is fast paced work, with little to no breaks or sick pay) that pose challenges to workers’ mental health and well-being, however, these challenges can also impact organisational outcomes.

The findings make a case to suggest mental health in this sector is poor, which has previously been found to be associated with organisational productivity through decreased efficiency and reduced capacity [36]. While the direct economic impact of poor mental health in the NTE hospitality sector is not clear [6], this paper established that poor mental health and poor well-being were associated with higher risk of poor work engagement and presenteeism as well as high turnover intentions. Presenteeism can lead to slower recovery from illness, increasing the risk of absenteeism in the future as well as health and safety risks in the workplace by increasing the risk of errors or accidents [37]. Furthermore, worker turnover leads to the loss of skilled workers and disrupted productivity for organisations [38]. With presenteeism (85%) and turnover intentions (40%) being prominent in this NTE hospitality sample, addressing poor mental health in the workforce can have important implications for workplace productivity and worker retention.

### Implications for workplace practice

Given the influence of poor mental health on organisational outcomes, NTE organisations have the opportunity to improve presenteeism and turnover intention outcomes through supporting worker mental health and well-being. A cross-sectional study on hotel workers found that focusing on resilience and creating a positive workplace environment could have the greatest impact on worker mental well-being [39]. Furthermore, perceived organisational support has been associated with higher job satisfaction and better mood, creating an opportunity for organisations to reduce turnover intentions [38]. A 2023 review estimated an overall small effect size associating adverse work conditions and risk of poor mental health [40]. This suggests that a preventative intervention, which typically has a small effect size, can mitigate more serious adverse (and costly) mental health and well-being outcomes further down the line [40].

Existing workplace interventions fail to provide practical mental health and well-being advice and policy guidelines for people in non-traditional workplaces [41], such as NTE hospitality workplaces. Many NTE roles, like bartending, event organising, or security, are not office-based roles with regularly scheduled hours, which means certain aspects of workplace mental health and well-being interventions, for example going on lunch time walks, may not speak to the needs of this sector. The development of NTE-specific interventions has the potential to mitigate long-term organisational costs, including employee sickness-related absences and staff turnover. Some efforts have been made to address this need in the UK with the launch of Thrive at Night [42] and Night Club [43]; there is an opportunity for future research to investigate whether these interventions are feasible to implement, which elements of these interventions are most needed, how effective they are in improving mental health and organisational outcomes, and whether they are transferable to different contexts.

### Strengths and limitations

This study is the first to our knowledge to utilise standardised mental health measures (WHO-5, PHQ-2, GAD-7, and the Single item of Burnout from the Physician Work Life Scale) in the UK NTE workforce.

A limitation of the present study is that only a quarter of the NTE-wide survey respondents worked exclusively at night, with most working a mix of day and night shifts. As some NTE organisations operate during daytime hours and people work multiple jobs, the NTE are hard to define. As there is currently no accepted criteria for defining NTE workers, the authors used the guidance of NTE stakeholders to define and recruit participants for this study. This is an understudied population and the criteria had to retain some flexibility. Ultimately, the research team had to rely on the participants self-identification as a “night-time worker.” The present research outlines that there are a number of challenges to mental health and well-being for those identifying as NTE workers; however, future research could investigate a more systematic way of defining NTE workers.

The sample size achieved for the NTE-wide open survey was approximately half of the target; data should be interpreted cautiously and replicated in future research. Reach and engagement required non-traditional approaches for this population. As this is largely a young work force, social media could be a powerful tool that could be better utilised to reach this target population. Additionally, access to a work email or formal online communication platforms were less common, meaning that more resources for in-person recruitment and relationship building with the community is essential for engaging NTE workers with research.

As this was an open survey, we were unable to collect reasons for non-participation. We chose not to use cookies or store IP addresses, for reasons of confidentiality and because workers may have completed the survey on shared devices. Therefore, it is possible that some respondents may not have been unique visitors, although this is unlikely given the low overall response rate. The convenience sample approach may have impacted the external validity of the findings; some groups may be under- or over-represented. For example, the survey sample contained a higher proportion of white British identity (83%) respondents compared to Bristol’s overall population (72%), which should be considered in the wider interpretation of the results [44]. We have no data on non-responders and so cannot estimate the risk of response bias, although efforts to minimise bias are described in study procedures. Nevertheless, the current sample is largely representative of Bristol’s NTE hospitality sector in terms of socio-demographic, job role, and organisational characteristics [45]. Such factors, including younger ages and working low paid roles within small-to-medium sized organisations at night, are fundamental features of the wider UK NTE. As a result, the current sample and findings will be broadly generalisable to other UK NTE contexts.

Due to the nature of surveys, data is self-reported which may introduce biases due to stigma and other factors. Furthermore, the survey was administered in English, which could have been a barrier to people who do not speak English or those who speak English as a second language.

## Conclusion

NTE workers frequently experience depression, anxiety, and burnout, impacting on work engagement. Work presenteeism is exceptionally high in this workforce, particularly in individuals with depression, anxiety or poor well-being. Alongside high turnover intentions, presenteeism is likely to result in considerable economic impact for employers. Further research should explore how and why poor mental health outcomes are commonly experienced in this sector through qualitative research. Exploring contextual experiences between different demographics, venue types, job roles and levels of responsibility will help identify key determinants and mechanisms of poor mental health, facilitating more tailored interventions. Future research should also investigate whether addressing mental health within the NTE hospitality sector could bring organisational benefits, such as reducing presenteeism and worker turnover, to NTE organisations. A mental health intervention may have the potential to help both worker mental health and well-being (risk of depression and anxiety) as well as organisational outcomes (work engagement, presenteeism and turnover intentions).

## Supplementary Information

Supplementary Material 1.

Supplementary Material 2.

Supplementary Material 3.

Supplementary Material 4.

## Data Availability

The data that support the findings of this study are available from the corresponding author upon reasonable request.
