# Supplementary material for: Characterising mental health and organisational outcomes in Bristol’s night time economy workforce: a cross-sectional online survey
Source: BMC Public Health. 2026 Jul 22;26:2990. doi: 10.1186/s12889-026-28676-4 (PMC13673694; doi:10.1186/s12889-026-28676-4)
Supplement: Supplementary file 1 [file 12889_2026_28676_MOESM1_ESM.docx]

Supplementary File. NTE-Wide Open Survey Measures

*The World Health Organisation – Five Well-being Index (WHO-5)*. Overall well-being was assessed using WHO-5 (World Health Organisation, 1998) questionnaire, which has five positively worded items rated on a six-point Likert-scale ranging from 0 = ‘at no time’ to 5 = ‘all of the time’. The WHO-5 is a validated and reliable tool for assessing self-reported well-being (Topp et al., 2015). The raw total scores range from 0 to 25, which were transformed to a scale of 0 to 100. A cut-off score of 50 is used to screen for well-being. Individuals/participants with scores ≤ 50 were considered to have poor well-being.

*Patient Health Questionnaire (PHQ-2).* Depression was assessed using the PHQ-2, which evaluates the presence of depressive symptoms over the past two weeks (Kroenke, Spitzer, & Williams, 2003). The short form version was used opposed to the long form due to concerns expressed by the public contributors that the overall survey was excessively long. The questionnaire consists of two items: (1) little interest or pleasure in doing things and (2) feeling down, depressed or hopeless. Each item is rated on a 4-point Likert scale, ranging from zero (not at all) to three (nearly every day), resulting in a total score ranging from zero to six. A cutoff score of 2 was used, with scores of 2 or higher indicating a positive screen for depression (Manea et al., 2016).

*Generalized Anxiety Disorder Scale (GAD-7)*. Anxiety over the past two weeks was assessed using the GAD-7, a seven-item questionnaire that estimates both the presence and intensity of anxiety (Spitzer et al., 2006). Each item is rated on a 4-point Likert-scale ranging from zero (not at all) to three (nearly every day).  The total scores range from zero to 21 with scores of 0-4, 5-9, 10-14 and 15-21 indicating minimal, mild, moderate and severe anxiety, respectively. GAD-7 has high reliability and validity for measuring anxiety (Löwe et al., 2008).

*Single item of Burnout from the Physician Work Life Scale.* The single-item burnout measure was used to assess burnout (West et al., 2009). The measure allows individuals to define burnout by responding to the question: *"Overall, based on your own definition of burnout, how would you rate your level of burnout?"* Responses were rated on a five-point ordinal scale from 1 indicating enjoyment of work with no signs of burnout to 5 representing severe burnout, where individuals may feel overwhelmed and consider making significant changes or seeking help. The responses were categorised into two groups: ≤ 2 scores indicate no burnout symptoms, and scores ≥ 3 indicate the presence of one or more symptoms. The single item Burnout scale is a validated and reliable tool for assessing burnout (West et al., 2012)

*Copenhagen Psychosocial Questionnaire, Version III (COPSOQ-III) short version.* To assess psychosocial risk factors at work, COPSOQ-III short version was used. The following factors of the short version (for workplaces) were included: Quantitative Demands, Work Pace, Influence at work, Recognition, Quality of Leadership, Mutual Trust Between Employees, Trust Regarding management, Justice at work, Social support from colleagues and Social support from supervisors. The questionnaire items were rated on a 5-point Likert scale ranging from zero (never/to a very small extent) to five (always/to a very large extent), with total scores being transformed into a 0-100 range. Higher scores indicate either more favourable or more problematic work conditions depending on the construct of the scale *(e.g. high “work demands” is negative, but high “job satisfaction” is positive).* This tool has a good internal reliability and has a Cronbach’s alpha of 0.72 (Burr et al., 2019)*.*

*9-item Utrecht Work Engagement Scale (UWES-9).* Work engagement was assessed using the UWES-9 scale (Schaufeli, Bakker, & Salanova, 2003). The scale consists of three dimensions of work engagement: vigour, dedication, and absorption with three items each which were rated on a 7-point Likert scale from zero (never) to six (always). The dedication sub-scale (3 items: DE2, DE3, DE4) was used in this study. Dedication refers to involvement and enthusiasm one feels toward their work. For the subscale dedication, mean scores were reported with higher mean scores indicating high dedication. UWES-9 scale has good validity and high rank-order stabilities for the work engagement factors (Seppälä et al., 2009).

*Turnover intentions.* Turnover intentions were assessed using a single global item used in a web-based survey with an employee population adapted from (Dolbier et al., 2005) and used in (Blake et al., 2020). The respondents responded either “yes” or “no” to a question adapted to fit NTE from the original question ‘‘Are you considering leaving or stopping your job because of mental-ill health?’’. The response was then analysed as a binary variable.

References:

Dolbier, C. L., Webster, J. A., McCalister, K. T., Mallon, M. W., & Steinhardt, M. A. (2005). Reliability and validity of a single-item measure of job satisfaction. *American Journal of Health Promotion*, *19*(3), 194-198.

Kroenke, K., Spitzer, R. L., & Williams, J. B. (2003). The Patient Health Questionnaire-2: validity of a two-item depression screener. *Medical care*, *41*(11), 1284-1292.

Löwe, B., Decker, O., Müller, S., Brähler, E., Schellberg, D., Herzog, W., & Herzberg, P. Y. (2008). Validation and standardization of the Generalized Anxiety Disorder Screener (GAD-7) in the general population. *Medical care*, *46*(3), 266-274.

Manea, L., Gilbody, S., Hewitt, C., North, A., Plummer, F., Richardson, R., ... & McMillan, D. (2016). Identifying depression with the PHQ-2: A diagnostic meta-analysis. *Journal of affective disorders*, *203*, 382-395.

Schaufeli, W. B., Bakker, A. B., & Salanova, M. (2003). Utrecht work engagement scale-9. *Educational and Psychological Measurement*.

Seppälä, P., Mauno, S., Feldt, T., Hakanen, J., Kinnunen, U., Tolvanen, A., & Schaufeli, W. (2009). The construct validity of the Utrecht Work Engagement Scale: Multisample and longitudinal evidence. *Journal of Happiness studies*, *10*, 459-481.

Topp, C. W., Østergaard, S. D., Søndergaard, S., & Bech, P. (2015). The WHO-5 Well-Being Index: a systematic review of the literature. *Psychotherapy and psychosomatics*, *84*(3), 167-176.

World Health Organization (1998). Wellbeing measures in primary health care/the depcare project. *Copenhagen: WHO Regional Office for Europe*, *46*(3), 0.
