# Supplementary material for: Characterising mental health and organisational outcomes in Bristol’s night time economy workforce: a cross-sectional online survey
Source: BMC Public Health. 2026 Jul 22;26:2990. doi: 10.1186/s12889-026-28676-4 (PMC13673694; doi:10.1186/s12889-026-28676-4)
Supplement: Supplementary file 3 [file 12889_2026_28676_MOESM3_ESM.docx]

**Supplemental File 3:** Sociodemographic Characteristics of the NTE-Wide Survey

|  | **Total**  *N =181* | **Percent (%)** |
| --- | --- | --- |
|  |  |  |
| **Gender**  Male  Female  Non-binary  Prefer not to say | 70  97  12  2 | *38.7*  *53.6*  *6.6*  *1.1* |
| **Age Category**  Under 25  25-34  35-44  Over 45 | 50  69  34  28 | *27.6*  *38.1*  *18.8*  *15.5* |
| **Highest Education**  GCSEs or equivalent  A / AS levels  NVQ or HND or HNC  College or University degree  Postgraduate degree  Other professional qualification  None of the above | 12  41  23  75  24  5  1 | *6.6*  *22.6*  *12.7*  *41.4*  *13.3*  *2.8*  *0.6* |
| **Student Status**  Yes  No | 27  154 | *14.9*  *85.1* |
| **Ethnicity**  White British  Mixed  Asian or Asian British  Black or Black British  Other | 150  5  3  3  20 | *82.9*  *2.8*  *1.6*  *1.6*  *11.1* |
| **Living Arrangement**  Alone  Living with partner  With family  With friends/housemates  Other | 24  49  28  73  7 | *13.3*  *27.1*  *15.4*  *40.3*  *3.9* |
| *- The table shows the frequencies in different sociodemographic categories (N and %).* | | |
