# Supplementary material for: Characterising mental health and organisational outcomes in Bristol’s night time economy workforce: a cross-sectional online survey
Source: BMC Public Health. 2026 Jul 22;26:2990. doi: 10.1186/s12889-026-28676-4 (PMC13673694; doi:10.1186/s12889-026-28676-4)
Supplement: Supplementary file 4 [file 12889_2026_28676_MOESM4_ESM.docx]

**Supplemental File 4:** Association between mental health, burnout and business-related outcomes

|  | **Work dedication** | |  | **Presenteeism** | |  | **Turnover intentions** | |
| --- | --- | --- | --- | --- | --- | --- | --- | --- |
|  | **Unadjusted**  **Mean Difference (95% CI)** | **Adjusted**  **Mean Difference (95% CI)** |  | **Unadjusted**  **OR (95% CI)** | **Adjusted**  **OR (95% CI)** |  | **Unadjusted**  **OR (95% CI)** | **Adjusted**  **OR (95% CI)** |
| **Depression**  No signs of depression  Signs of depression | Ref  - 0.72 [-1.0, -0.44] | Ref  - 0.62 [-0.91, -0.33] |  | Ref  3.28 [1.45,7.73] | Ref  3.44 [1.40, 8.83] |  | Ref  2.60 [1.18, 5.96] | Ref  2.70 [1.14, 6.67] |
| **Anxiety**  No/minimal anxiety  Some level of anxiety | Ref  -0.47 [-0.77, -0.18] | Ref  -0.32 [-0.64, -0.00] |  | Ref  3.49 [1.53,8.22] | Ref  4.00 [1.59, 10.65] |  | Ref  3.52 [1.59, 8.20] | Ref  3.53 [1.44, 9.23] |
| **Wellbeing**  Good wellbeing  Poor wellbeing | Ref  -0.85 [-1.12, -0.57] | Ref  -0.79 [-1.06, -0.51] |  | Ref  1.84 [0.82,4.19] | Ref  1.68 [0.71, 4.07] |  | Ref  4.63 [2.15, 10.50] | Ref  4.84 [2.12, 11.74] |
| **Burnout**  No burnout signs  1 or more burnout signs | Ref  -0.67 [-0.94, -0.39] | Ref  -0.60 [-0.88, -0.32] |  | Ref  3.01 [1.31,7.41] | Ref  2.85 [1.17, 7.36] |  | Ref  6.89 [3.05, 16.95] | Ref  8.45 [3.39, 23.57] |
| Significant results are those where the confidence intervals don’t include 1 for ORs and 0 for mean difference.  Adjusted for gender, highest education, age category, and ethnicity. For the regression models due to small proportions in some categories, gender was collapsed into three categories (male, female and other) “prefer not to say” was merged with “non-binary” category. Highest education was collapsed into three categories, Low ("None of the above", "GCSEs or equivalent"), Medium ("A/AS levels", "NVQ or HND or HNC", "Other professional qualification") and High ("College or University degree", "Postgraduate degree"). Ethnicity was collapsed into two categories White (White British) and Ethnic minority (all other categories).  CI – confidence interval, OR – Odds Ratio | | | | | | | | |
